# Book Reviews, Etc.

**Published:** 1895-12

**Authors:** 


### Book Reviews, Etc.

---

Taylor on Venereal Diseases: The Pathology and Treatment of Venereal Diseases. By Robert W. Taylor, A. M., M. D., Clinical Professor of Venereal Diseases in the College of Physicians and Surgeons, New York. In one very handsome octavo volume, 1002 pages, with 230 engravings and 7 colored plates. Cloth, $5.50; leather, $6.50. Philadelphia: Lea Brothers & Co., Publishers, 1895.

This book represents an entire revision of the author's former work in connection with Dr. Freeman L. Bumstead. The enormous addition to our knowledge of venereal diseases in the last decade has effected every branch of the subject, so that a text-book of ten years ago, has only a historical value. Dr. Taylor's extensive experience as a lecturer and a clinician renders him peculiarly suited to collect and present in a succinct and authoritative manner these advances in venereal pathology and treatment.

His attitude toward the much discussed "latent" gonorrhœa in the female, is one of disbelief, a view that quite coincides with the experience of many genito-urinary surgeons, but is not generally shared by gynæcologists. The volume is profusely illustrated.

Materia Medica and Therapeutics: A Practical Treatise with Especial Reference to the Clinical Application of Drugs. By John V. Shoemaker, A. M., M. D., LL.D., Professor of Materia Medica, Pharmacology, Therapeutics, and Clinical Medicine, and Clinical Professor of Diseases of the Skin in the Medico-Chirurgical College of Philadelphia; Physician to the Medico-Chirurgical Hospital, Philadelphia, etc., etc. Third edition. Thoroughly revised, reset with new type and printed from new electrotype plates. Royal octavo, pages ix, 1108. Extra cloth, $5.00 net; sheep, $5.75 net. Philadelphia: The F. A. Davis Co., Publishers, 1914 and 1916 Cherry Street.

The third edition of this valuable work combines in one the two volumes formerly published separately. The progress of this province of medicine has been duly recorded and new remedies that have proven of undoubted value receive extended notice. Following this, there are descriptions of acetanalid, dermatol, creosote and its derivatives, new naphthol and phenol compounds, etc. The therapy of various morbid conditions has received an impartial presentation.

The work is sufficiently known and appreciated as to require no introduction to our readers.

**PRACTICAL URANALYSIS AND URINARY DIAGNOSIS: A MANUAL FOR THE USE OF PHYSICIANS, SURGEONS AND STUDENTS. By CHARLES W. PURDY, M. D., Queen's University; Fellow of the Royal College of Physicians and Surgeons, Kingston; Professor of Urology and Urinary Diagnosis at the Chicago Post-Graduate Medical School; Author of "Bright's Disease and Allied Affections of the Kidneys"; also of "Diabetes: Its Causes, Symptoms, and Treatment." SECOND, REVISED, EDITION. With Numerous illustrations, including photo-engravings and colored plates. In one crown octavo volume, 360 pages, in extra cloth, $2.50 net. Philadelphia: The F. A. Davis Co., Publishers: 1914 and 1916 Cherry Street.**

As a text-book and book of reference for the practitioner, this work is without an equal. So far as we are aware, it is the most comprehensive book yet published upon the subject of Urinary Analysis, and contains much information which must usually be sought for in works upon Pathology, Chemistry, Physiology, etc. While the author goes over the numerous urinary tests, yet he distinctly advocates the use of only such tests as can be relied upon, and points out the sources of error so common in Urinary Analysis. The second portion of the work takes up Urinary Diagnosis and shows how the various pathological conditions of the economy may be diagnosticated by a study of the changes in the urine. The work is well illustrated and printed in good style.

